# L-DNA-Based Melt Analysis Enables Within-Sample Validation of PCR Products

**DOI:** 10.1021/acs.analchem.4c01611

**Published:** 2024-07-08

**Authors:** Nicole A. Malofsky, Dalton J. Nelson, Megan E. Pask, Frederick R. Haselton

**Affiliations:** † Department of Biomedical Engineering, Vanderbilt University, Nashville, Tennessee 37235, United States

## Abstract

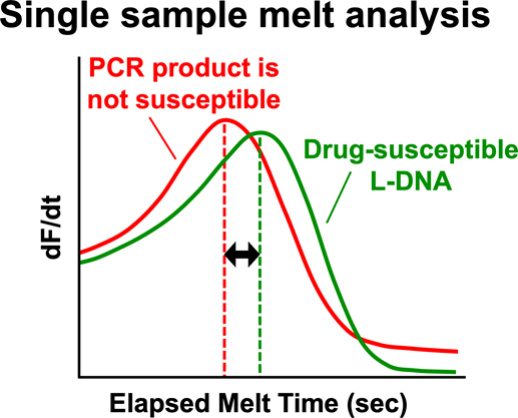

The melt analysis feature in most real-time polymerase chain reaction (PCR) instruments is a simple method for determining if expected or unexpected products are present. High-resolution melt (HRM) analysis seeks to improve the precision of melt temperature measurements for better PCR product sequence characterization. In the area of tuberculosis (TB) drug susceptibility screening, sequencing has shown that a single base change can be sufficient to make a first-line TB drug ineffective. In this study, a reagent-based calibration strategy based on synthetic left-handed (L)-DNA, designated LHRM, was developed to confirm validation of a PCR product with single base resolution. To test this approach, a constant amount of a double-stranded L-DNA melt comparator was added to each sample and used as a within-sample melt standard. The performance of LHRM and standard HRM was used to classify PCR products as drug-susceptible or not drug-susceptible with a test bed of nine synthetic *katG* variants, each containing single or multiple base mutations that are known to confer resistance to the first-line TB drug isoniazid (INH). LHRM achieved comparable classification to standard HRM relying only on within-sample melt differences between L-DNA and the unknown PCR product. Using a state-of-the-art calibrated instrument and multiple sample classification analysis, standard HRM was performed at 66.7% sensitivity and 98.8% specificity. Single sample analysis incorporating L-DNA for reagent-based calibration into every sample maintained high performance at 77.8% sensitivity and 98.7% specificity. LHRM shows promise as a high-resolution single sample method for validating PCR products in applications where the expected sequence is known.

## Introduction

Sequencing of tuberculosis (TB) drug-resistant strains has shown that many drug-resistant variants have one or more single nucleotide polymorphisms (SNPs) often clustered in contiguous regions of the drug-resistant genome.^1−3^ These resistance-related characteristic changes provide potential biomarkers for drug treatment decisions. However, requiring sequencing for every positive TB sample as part of a clinical treatment algorithm remains cost-prohibitive in many resource-constrained settings.

When the presence of drug-susceptible cases is high, a follow-on test to confirm drug susceptibility provides a pragmatic first step in a clinical treatment algorithm. The goal is to confirm drug susceptibility for the majority of samples and focus the limited resources on more complex testing for the small number of cases that are not drug-susceptible.

Based on known TB sequencing data, amplification-based susceptibility testing has been used to characterize samples and inform the drug treatment algorithm. These approaches are divided into two categories, direct or indirect testing. Direct testing confirms the presence of one or more specific SNPs that make the strain untreatable by a particular drug. Alternatively, indirect testing, based on the SNP clustering observation, broadly seeks to confirm the presence of the nonmutated, susceptible sequence that makes the strain treatable by a particular drug.

In the presence of multiple SNPs that independently confer resistance, the former SNP-targeted approach requires either implementation of a separate test for each SNP or a multiplexed design including all SNPs. Although this direct testing approach shows promise for particular TB strains of concern,^4,5^ it remains difficult to scale as the number of drug-resistant SNPs increases.

The latter cluster-based strategy for susceptibility testing is based on polymerase chain reaction (PCR) detection. This approach seeks to validate that the PCR product has the known drug-susceptible sequence by melt analysis. Melt analysis is based on the observation that any given double-stranded DNA sequence dissociates at a characteristic melt temperature (*T*_m_). This property is used to compare the melt temperature of an unknown PCR product to the characteristic melt temperature of the known drug-susceptible wild-type sequence. Any shift from this wild-type melt temperature implies that the unknown test sample contains one or more SNPs. Melt analysis capitalizes on the hardware capabilities of PCR instrumentation and is often available in real-time PCR instruments. Some real-time instruments also offer high-resolution melt (HRM) capabilities by including a temperature calibration feature. Because of many variables that affect the melt properties, standard HRM classifies an unknown sample by comparing the melt temperature of the unknown PCR product to the melt temperature of a known PCR product,^4−10^ usually included in as additional samples in the assay. The requirement for instrument calibration to enable the comparison of two or more samples is a major source of complexity in these approaches. In the case of current PCR and melt-based TB drug susceptibility tests Xpert MTB/RIF Ultra^11^ and Xpert MTB/XDR,^12^ unknown samples are compared to an algorithm-based reference library of *T*_m_ signatures from a set of known mutations.^13^ Proprietary designs around this relatively expensive technology, however, continue to limit its utility, particularly where it is most needed. Since a limited number of point-of-care diagnostics currently offer low-cost TB drug resistance testing by HRM,^14−17^ there is a demonstrated need for simpler validation of drug susceptibility in the TB treatment algorithm using more widely available real-time PCR instruments.

In this report, a potential single sample approach based on reagent-based calibration is proposed to simplify HRM and avoid the requirement for multiple sample comparisons in every assay. In this design, left helical L-DNA is added to every sample as a standard melt comparator. The approach is based on the assumption that both double-stranded L-DNA additive and double-stranded D-DNA PCR product in the same well are affected by hybridization melt characteristics in the same way. If the melt characteristics of the L-DNA additive and the D-DNA from the PCR amplicon of a drug-susceptible sample are set identical, any difference in melt characteristics between L-DNA and an unknown PCR product is attributed to a change in PCR product sequence. In other words, the drug-susceptible reference sequence is included for comparison to the sample PCR amplicon, not in a separate well as D-DNA, but rather within each sample as L-DNA.

Two key features of L-DNA support the feasibility of this approach. First, published reports suggest that L-DNA does not interfere or participate in PCR reactions^18−23^ and has been employed in applications where it does not interact with normal biological processes, such as intracellular biosensing^24−27^ and PCR control.^22,23^ Second, several reports suggest that L-DNA and naturally occurring D-DNA with identical sequences have identical melt characteristics,^24,28,29^ suggesting that matching melt characteristics should be possible.

Performance of L-DNA-based HRM (LHRM) was compared to standard HRM using a state-of-the-art HRM instrument for both methods. The assays were applied to drug susceptibility screening for isoniazid (INH), a first-line prodrug therapeutic for TB. The internal comparator L-DNA was synthesized as a 56-nucelotide sequence from the drug-susceptible TB *katG* gene where over 250 INH-resistance-related mutations are clustered.^30^ The nine synthetic variants were selected to provide product validation challenges that ranged from relatively easy, due to multibase mutations, to very difficult, due to only a single base mutation. PCR products of these synthetic targets were classified as drug-susceptible or not drug-susceptible and used to compare the LHRM and standard HRM methods.

## Experimental Section

### DNA Oligonucleotide Design

The melt analysis test bed was developed using the drug-susceptible TB *katG* gene.^30^ A single primer set was designed to cover the most prevalent variant S315T found in 94% of INH-resistant clinical isolates^31,32^ and a subset of the many single or multibase variants in the neighboring region that also confer INH resistance.^1,32−35^ Single-stranded PCR targets were synthesized with D-DNA sequences of drug-susceptible wild-type *katG* (H37Rv: 2153889–2156111) and nine clinically relevant drug-resistant *katG* mutants (Table 1). The selected variants included a range of melt differences from wild type, offering both easy and challenging drug susceptibility classification cases. The theoretical *T*_m_ spread of variants was 2.8 °C based on a nearest neighbor oligonucleotide calculator^36^ with automated settings. Detailed information on the DNA oligonucleotide sequences used in these studies is shown in Table S1. All DNA oligonucleotides employed for development and testing of the assay were synthesized by Integrated DNA Technologies (Coralville, Iowa, USA) or biomers.net (Ulm, Baden-Württemberg, Germany).

**Table 1:**
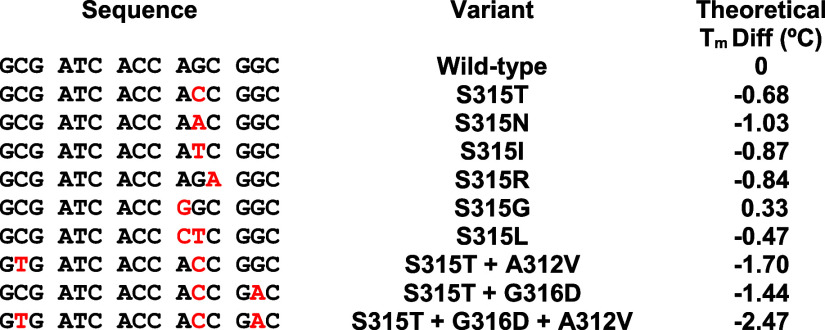
Sequences (Written 5′-to-3′) of the Drug-Susceptible Wild-Type *katG* and Nine *katG* Variants^a^

a Each variant has INH-resistance-related mutations (red) that induce theoretical melt differences (*T*_m_ diff.) from wild-type (right column).

### Standard HRM Approach

The standard HRM approach for drug susceptibility screening is based on a two-sample comparison of *T*_m_’s between an unknown PCR product and a known drug-susceptible PCR product (Figure S1). Reactions were performed in the QuantStudio 5 real-time PCR thermal cycler (Thermo Fisher Scientific #A28137). This highly capable instrument was selected to facilitate standard HRM performance as a state-of-the-art comparison method for LHRM.^37^Reactions had a 20 μL final volume containing 1X of SensiFAST Probe No-ROX Kit (Bioline #BIO-86005), 1X LCGreen Plus (BioFire Defense, LLC #BCHM-ASY-005), and 250 nM of each *katG*-specific primer (MEP176 and MEP177). Each target sample contained a final concentration of wild-type (MEP183) or mutant (MEP184–189,197–199) single-stranded DNA target at 2 × 10^6^ copies per reaction. An example of a standard HRM reaction setup is outlined in Table S2. PCR reactions were initiated with a 95 °C hold for 2 min followed by 40 cycles of 95 °C for 5 s and 59 °C for 20 s. A HRM was performed immediately following PCR by annealing 95–50 °C at 0.1 °C/s followed by melting 65–95 °C at 0.025 °C/s (continuous acquisition mode).^38−41^ Double-stranded DNA PCR product fluorescence was monitored during PCR and during the melt reaction using LCGreen Plus on the green optical channel (excitation 470 ± 15/emission 520 ± 15). Complete details are included in Supporting Information (see page S3).^42^

### Standard HRM Analysis and Statistics

PCR quantification cycle (*C*_q_) was determined with the QuantStudio 5 Design and Analysis Software. Nonamplifying samples did not report C_q_ and were excluded from the data analysis. Amplifying samples with *C*_q_ over 35 were excluded from the data analysis because they did not achieve the PCR plateau phase. Representative PCR amplification curves of samples are included in Figure S11. *T*_m_ was calculated with the proprietary QuantStudio 5 Design and Analysis Software based on the first derivative of fluorescence with respect to temperature. Based on *T*_m_ analysis of all samples, *T*_m_ cutoff points were established to maximize test specificity when classifying standard HRM analyzed samples as drug-susceptible or not. Specificity was maximized to decrease the false-positive rate, i.e., decrease the misdiagnosis of variant samples as drug-susceptible. This maximized specificity strategy is often used for HRM classification of TB samples with drug resistance.^10,43,44^ Each test sample was individually classified. A sample was classified as drug-susceptible when PCR product *T*_m_ was within the drug-susceptible *T*_m_ cutoff range of 82.4 and 82.5 °C. Since true positives are known, standard HRM was assessed for its sensitivity and specificity using this *T*_m_ cutoff range to classify drug susceptibility among 9 true drug-susceptible samples (*n* = 3 trials of wild-type in triplicate) and 81 true not drug-susceptible samples (*n* = 3 trials of 9 variant types in triplicate). In the experiment testing heating variability, significance was evaluated using *T*_m_ comparison (unpaired *t* test, significance level of α = 0.95) of 96-well plate quadrants of S315T as compared to wild-type (*n* = 1 trial with 24 replicates per sample type). All statistics were performed in Microsoft Excel 2022 except for the sensitivity and specificity analysis that was performed in Python. Complete details are included in Supporting Information (see page S3).

### LHRM Approach

LHRM for drug susceptibility screening is based on elapsed melt time (*t*_m_) comparison between an unknown PCR product and a drug-susceptible L-DNA comparator within a single sample (Figure S2). To ensure a fair comparison between LHRM and standard HRM, both methods were tested using the same QuantStudio 5 instrument. LHRM used identical PCR cycling, PCR fluorescence monitoring, PCR quantification, melt reaction cycling, reaction loading placement, and heating variability test setup as standard HRM. LHRM statistics were identical to that of standard HRM, except for a data subset testing heating variability. Key changes from standard HRM are the inclusion of an additional reagent (L-DNA), monitoring melt reaction fluorescence on a second optical channel, and analysis of fluorescence changes as a function of time from the start of the QuantStudio 5 continuous mode melt instead of melt temperature provided by the instrument’s calibration.

A double-stranded L-DNA drug-susceptible comparator was synthesized using left helical enantiomeric DNA bases (i.e., L-DNA)^45^ with an identical sequence to the known drug-susceptible *katG* sequence. The 56-base L-DNA was synthesized with the same length and sequence as the drug-susceptible PCR amplicon. The double-stranded L-DNA was end-labeled with Texas Red (TXR) fluorophore and Black Hole Quencher 2 (BHQ2) quencher to monitor its behavior during melting on the orange fluorescence channel (excitation 580 ± 10/emission 623 ± 14). Detailed information on the L-DNA oligonucleotide sequences used in these studies is shown in Table S1. LHRM reactions included 2 μL of L-DNA mix with final copy counts of 1 × 10^11^ copies TXR-labeled forward strand L-DNA (23FEB_katGf56_TXR) and 3 × 10^11^ copies BHQ2-labeled reverse strand L-DNA (23FEB_katG_56_Rcmp+5_BHQ2) per reaction. An example reaction setup containing the L-DNA additive is outlined in Table S3.

To ensure identical melt characteristics of D-DNA and end-labeled L-DNA, additional experiments were performed varying L-DNA strand concentration and strand ratio. In experiments varying L-DNA strand concentrations, reaction component deviations included 100 nM final concentration of each *katG*-specific primer and 1 × 10^11^, 2 × 10^11^, and 4 × 10^11^ copies of L-DNA strands (forward and reverse) per reaction. In experiments varying L-DNA forward to reverse strand ratio, reaction component deviations included 100 nM final concentration of each *katG*-specific primer and 2 μL of L-DNA mix at 1:1, 1:2, and 1:3 ratios of forward to reverse strands for final L-DNA copy numbers of 1 × 10^11^ copies of forward strand plus 1 × 10^11^, 2 × 10^11^, and 3 × 10^11^ copies of reverse strand, respectively. Linear interpolation of three different L-DNA strand ratios was used to determine the relationship between copies of L-DNA reverse strands per reaction and L-DNA melt measurement. The L-DNA reverse strand copy number with a melt measurement matching that of wild-type PCR product was selected. Complete details are included in Supporting Information (see page S4).

### LHRM Analysis and Statistics

Representative PCR amplification curves of samples containing L-DNA are included in Figure S12. Elapsed melt time (*t*_m_) was calculated from the second degree Savitsky–Golay polynomials^46^ at each point (performed in MATLAB 2023A) based on the first derivative of fluorescence with respect to elapsed melt time. Elapsed melt time is a means of *T*_m_ reporting derived from the uncalibrated QuantStudio 5 raw data. Here, *t*_m_ is defined as the elapsed melt time (in seconds) to reach the maximum derivative of fluorescence with respect to elapsed melt time. Significant differences between wild-type PCR product and L-DNA within each sample were assessed using paired *t* tests (of *t*_m_) with a significance level of α = 0.95 (*n* = 3 trials in triplicate). Test samples were classified as drug-susceptible when sample *t*_m_ difference was zero. LHRM classification criteria are based on our assumption that L-DNA and PCR product melt characteristics are identical if and only if their sequences match. Specificity was maximized to decrease the false-positive rate. Since true positives are known, LHRM was assessed for its sensitivity and specificity using a *t*_m_ difference of zero to classify drug susceptibility among 9 true drug-susceptible samples (*n* = 3 trials of wild type in triplicate) and 79 true not drug-susceptible samples (*n* = 3 trials of 9 variant types in triplicate, except variant S315T+G316D+A312V which had one trial with a single replicate due to *C*_q_ exclusion).

To directly compare time-based LHRM analysis within a single sample and temperature-based standard HRM analysis between samples, L-DNA-containing samples were also analyzed using standard HRM analysis. Sample *T*_m_ was calculated with the proprietary QuantStudio 5 Design and Analysis Software. Based on *T*_m_ analysis of all samples, *T*_m_ cutoff points were established to maximize test specificity when classifying each test sample as drug-susceptible or not. A sample was classified as drug-susceptible when PCR product *T*_m_ was within the drug-susceptible *T*_m_ cutoff range of 82.4 and 82.5 °C. Since true positives are known, classification sensitivity and specificity were assessed using this *T*_m_ cutoff range to classify drug susceptibility among 9 true drug-susceptible samples (*n* = 3 trials of wild type in triplicate) and 79 true not drug-susceptible samples (*n* = 3 trials of 9 variant types in triplicate, except variant S315T+G316D+A312V of one trial with a single replicate due to *C*_q_ exclusion).

Alternative strategies exist to establish drug-susceptible classification cutoff points for HRM analysis, and this was explored in Supporting Information (see pages S10–S11). This supplemental work used a maximized Youden J Statistic^47^ to establish drug-susceptible classification cutoff points for the same data sets across standard HRM and LHRM analysis strategies (see pages S10–S11). This alternative cutoff strategy generally improved sensitivity and decreased specificity.

In the experiment testing heating variability, significance was evaluated using melt measurement comparison (Mann–Whitney *U* test, significance level of α = 0.95) of 96-well plate quadrants of S315T as compared to wild type (*n* = 1 trial with 24 replicates). The heating variability Mann–Whitney *U* test was performed twice, once using *T*_m_ as the melt measurement and once using *t*_m_ difference as the melt measurement. All statistics were performed in Microsoft Excel 2022 except for the sensitivity and specificity analysis that was performed in Python. Complete details are included in Supporting Information (see pages S4–S5).

## Results and Discussion

**Figure 1:**
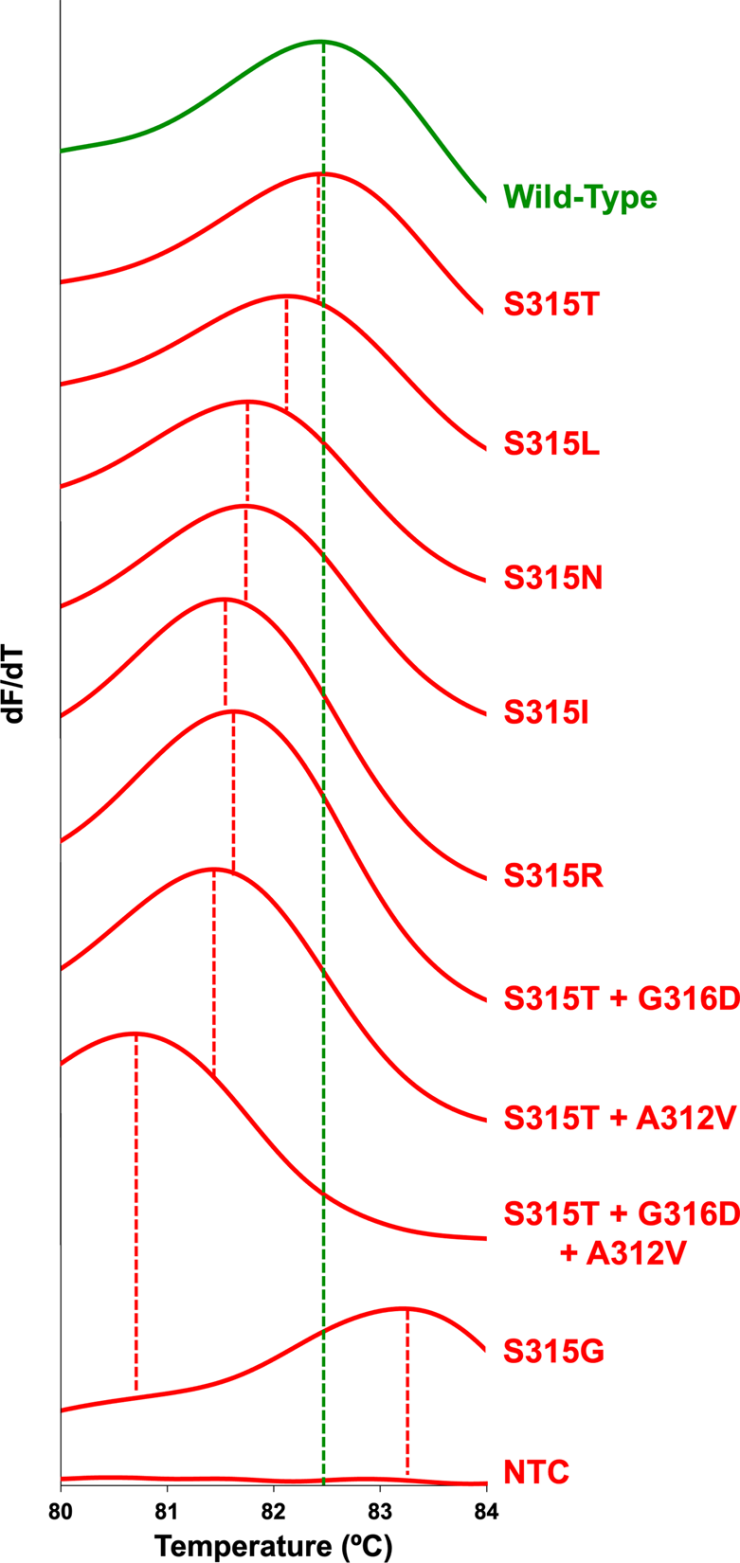
Multisample comparison of *T*_m_ obtained from standard high-resolution melt of 11 representative samples containing wild-type (green), one of nine variants (red), or no template control polymerase chain reaction products. Dashed vertical lines indicate the *T*_m_ for each sample on the horizontal axis.

**Figure 2:**
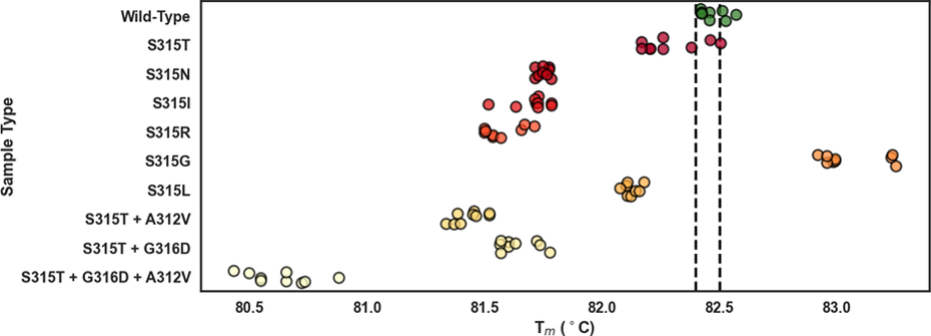
Polymerase chain reaction product melt temperatures of samples analyzed by standard high-resolution melt across wild-type (green) and nine variants. Samples were classified as drug-susceptible or not by comparing sample *T*_m_ to the drug-susceptible *T*_m_ cutoff range of 82.4 and 82.5 °C (indicated by dashed black lines). Each point represents an individual test sample.

An initial classification of the test variants by standard HRM confirmed that classification was successful for most variants but that the small melt difference in the single base mutation S315T was near the limits of this calibrated instrument approach. As predicted by the theoretical melt differences (Table 1), all variant samples analyzed by standard HRM had lower melt temperatures compared to the known wild-type samples, except S315G which had a higher melt temperature (Figure 1). The nine selected variants had a *T*_m_ spread of 2.43 °C, offering both easy and challenging classification cases against wild type (Figure 1). Average sample *T*_m_’s and *T*_m_ differences are reported in Table S4. Standard HRM correctly classified 6/9 wild-type *katG* samples as drug-susceptible and 80/81 variant samples as not drug-susceptible; the most clinically prevalent variant S315T was misclassified once. Although S315G has the smallest theoretical melt difference (0.33 °C) from wild type and was initially thought to be the most difficult variant to correctly classify, S315T was experimentally the most difficult case because under the sample salt conditions, it induced the smallest melt difference (−0.18 °C) from wild type among all nine variants. Standard HRM relied on *T*_m_ analysis of data from multiple samples to form the drug-susceptible classification *T*_m_ cutoff range of 82.4 to 82.5 °C. Using a state-of-the-art calibrated instrument, standard HRM performed at 66.7% sensitivity and 98.8% specificity when classifying drug susceptibility. Sample classification accuracy and relationships between sample type *T*_m_’s are shown in Figure 2. In particular, standard HRM misclassification is illustrated by three wild-type samples above the upper drug-susceptible cutoff range and one S315T sample within the drug-susceptible cutoff range.

This S315T classification error was thought to be due to instrument heating variability that has been observed in many plate-based real-time PCR instruments.^48^ Nonuniform heating limits *T*_m_ comparison accuracy^48^ and is only partially compensated by instrument calibration.^37^ In a direct test of this, the QuantStudio 5 was found to exhibit heating variability across the 96-well plate (Figure S3) and this impacted standard HRM classification of the most difficult case, S315T. Standard HRM failed to distinguish between wild type and S315T when using PCR product melt characteristics since there was no significant difference between wild-type and S315T sample *T*_m_’s (*p* > 0.05, unpaired *t* test, 24 replicates per sample type). The 24 identical samples exhibited thermal edge effects of generally higher *T*_m_’s and zone-based *T*_m_’s for two samples in shared heating elements, with trends holding for both wild-type and S315T sample types (Figure S3). Specifically, wild-type average PCR product *T*_m_ (mean ± SD) was 82.46 ± 0.08 °C across all wells and 82.53 ± 0.07 °C across edge wells. S315T average PCR product *T*_m_ (mean ± SD) was 82.42 ± 0.10 °C across all wells and 82.50 ± 0.09 °C across edge wells. The heating variability of the QuantStudio 5 (Figure S3) is presumably due to variations in the instrument’s six independent temperature heating zones (pairwise vertical columns)^37^ that are not completely corrected by the instrument calibration procedures.

We used this simple experimental design to test the initial feasibility of the L-DNA approach. Repeating this heating experiment but incorporating L-DNA into each sample did not change the experimental outcome. The approach still failed to distinguish between wild type and S315T because there was no statistical difference between PCR product *T*_m_’s (*p* > 0.05, Mann–Whitney *U* test, 24 replicates per sample type, column 2 in Table S7). These results confirmed that the addition of L-DNA itself does not affect the between-sample analysis based on temperature and indirectly supports the initial assumption that L-DNA does not interfere with the PCR reaction. However, reanalysis using PCR product to L-DNA melt time differences did successfully overcome effects from nonuniform heating and distinguished between variant S315T and wild type. The additional L-DNA elapsed time data in this experimental design were used to show that a reanalysis incorporating a within-sample melt difference between L-DNA and PCR product distinguishes between these two sequences with a single base difference. Using *t*_m_ differences, there was a significant difference between quadrants of wild-type and S315T samples (*p* < 0.05, Mann–Whitney *U* test, 24 replicates per sample type, column 4 in Table S7).

Due to the success of the limited data set employing L-DNA, the full variant test bed (Table 1) previously performed (Figure 2) was run incorporating an L-DNA comparator into every sample. These data were analyzed by two different methods: standard HRM analysis using temperature-based melt characteristics between PCR products of multiple samples and LHRM analysis using time-based melt differences between the PCR product and L-DNA comparator within each sample.

**Figure 3:**
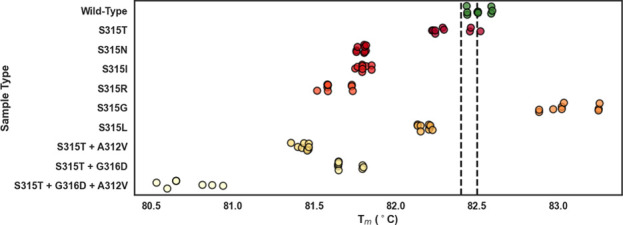
Polymerase chain reaction product melt temperatures of samples containing an L-DNA comparator in every sample but analyzed by standard HRM across wild-type (green) and nine variants. Samples were classified as drug-susceptible or not by comparing sample *T*_m_ to the drug-susceptible *T*_m_ cutoff range of 82.4 and 82.5 °C (indicated by dashed black lines).

As shown in Figure 3, standard HRM analysis classification accuracy of L-DNA-containing samples was similar to the original standard HRM experiment without L-DNA (Figure 2). Average sample *T*_m_’s and *T*_m_ differences are reported in Table S5. 3/9 wild-type *katG* samples were correctly classified as drug-susceptible and 77/79 variant samples were correctly classified as not drug-susceptible. Standard HRM misclassification is illustrated by six wild-type samples above the upper drug-susceptible cutoff range and two S315T samples within the drug-susceptible cutoff range (Figure 3). Standard HRM analysis of L-DNA-containing samples performed at 33.3% sensitivity and 97.5% specificity when classifying drug susceptibility. Samples containing L-DNA and analyzed by standard HRM had decreased sensitivity and comparable specificity metrics as compared to samples without L-DNA and analyzed by standard HRM.

**Figure 4:**
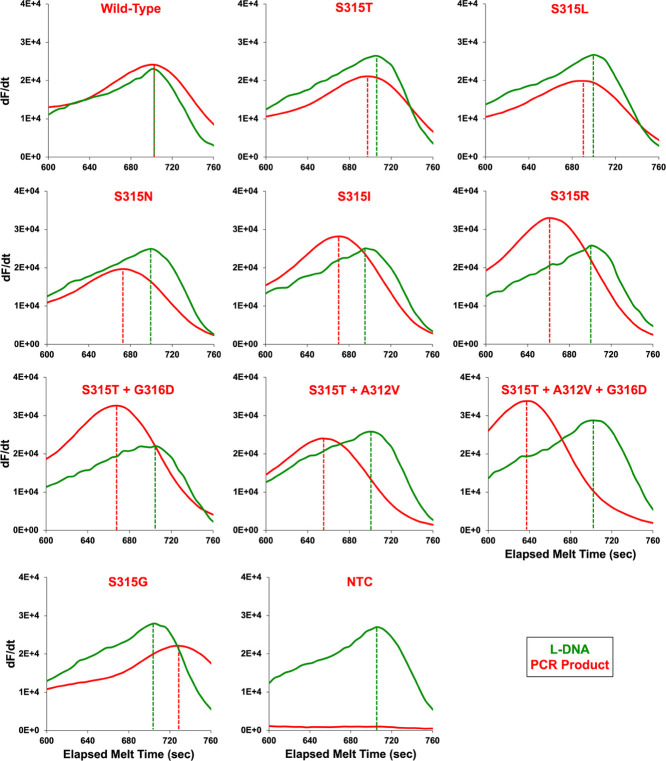
Within-sample comparison of *t*_m_ obtained from LHRM for 11 representative samples containing both wild-type L-DNA (green) and wild-type, variant, or NTC PCR products (red). Dashed vertical lines indicate the two *t*_m_’s within each sample on the horizontal axis.

Samples containing L-DNA were reanalyzed via LHRM analysis. The LHRM and standard HRM analysis strategies resulted in similar melt behavior across the wild-type and full variant test bed (Figure S4). Using LHRM, all variant samples had lower elapsed melt times compared to the drug-susceptible comparator, except S315G which had a higher elapsed melt time (Figure 4). L-DNA and DNA PCR product had nearly identical melt characteristics when the sequences matched (wild-type *katG*) but differed if there was a sequence mismatch (*katG* variants) (Figure 4 and Table S6). Using melt time differences between PCR product and L-DNA within each sample, LHRM correctly classified 7/9 wild-type *katG* samples as drug-susceptible and 78/79 variant samples as not drug-susceptible.

**Figure 5:**
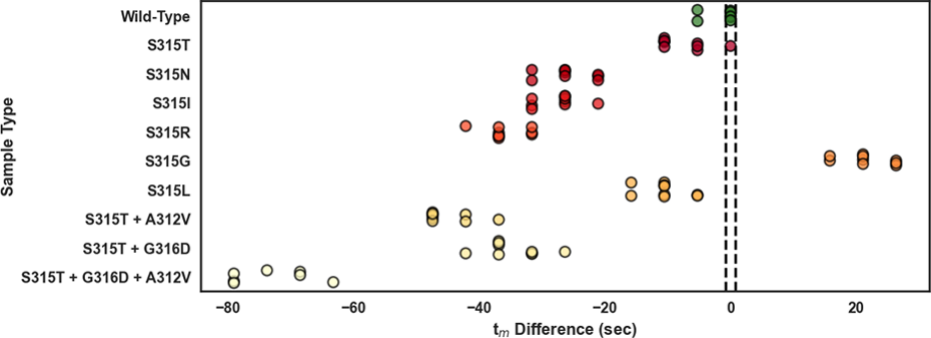
Within-sample *t*_m_ differences of samples containing an L-DNA comparator in every sample and analyzed by LHRM across wild-type (green) and nine variants. Samples were classified as drug-susceptible or not by comparing sample *t*_m_ difference to the drug-susceptible classification criteria of *t*_m_ difference = 0 (indicated by dashed black lines).

Unlike standard HRM, LHRM drug-susceptible classification criteria were established without relying on analysis of data from multiple samples. Instead, LHRM classified samples as drug-susceptible when a sample’s *t*_m_ difference was zero. This simple classification strategy enabled single sample classification without requiring data from other samples. LHRM performed at 77.8% sensitivity and 98.7% specificity when classifying drug susceptibility. Sample classification accuracy and relationships between sample type *t*_m_ differences are illustrated in Figure 5, which can be directly compared to Figure 3. In particular, LHRM misclassification is illustrated by two wild-type samples below the drug-susceptible cutoff and one S315T sample within the drug-susceptible cutoff (Figure 5). S315T was the only variant misclassified by LHRM analysis, as similarly observed in standard HRM analysis classification (Figure 3). LHRM correctly classified drug susceptibility with specificity very similar to and with improved sensitivity over standard HRM analyzed samples. This trend held for LHRM as compared to standard analyzed samples with and without L-DNA. Notably, LHRM accomplished high success metrics without requiring multiple sample classification data and without relying on temperature-based melt reporting determined by instrument calibration.

Heating variation was identified as a major methodological artifact that reduced classification performance for S315T by HRM (Figure S3). While no other methodological artifacts were identified in HRM testing, it was speculated that some types of sample preparation errors could introduce systematic hybridization changes that could also be corrected using L-DNA. Possible errors may include culture media carryover, extraction errors, kit-to-kit master mix differences, or sample-to-sample salt concentration variability resulting from reagent pipetting errors.^8^ A contrived study of salt differences is included in Supporting Information (see page S12) to demonstrate an example of how LHRM can correct for these types of errors. In particular, this supplemental study verified that a sample’s salt content has a detrimental potential impact on classification accuracy and that within-assay differences can be overcome by LHRM to ultimately restore classification capabilities.

Three key features are critical for the success of the LHRM approach. First, LHRM reactions must include the same amount of double-stranded L-DNA in every sample to provide a signature comparator hybridization event. Constant L-DNA concentration in every sample was maintained by adding L-DNA from a stock into the master mix. Since L-DNA and the PCR product hybridization events were changed by heating (Figure S3) and salt variation errors (see Supporting Information, page S12) in the same way, the L-DNA to PCR product melt difference corrected for any artifact-induced melt shifts. Small mutation-induced melt shifts in the PCR product were then detectable due to the reduction in between-sample errors and ultimately facilitated LHRM classification performance with specificity very similar to and improved sensitivity over the standard method.

**Figure 6:**
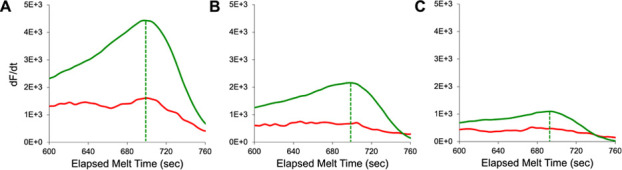
Melt curve derivatives for LHRM samples with 1:1 double-stranded L-DNA at (A) 4 × 10^11^, (B) 2 × 10^11^, and (C) 1 × 10^11^ copies per strand (forward and reverse) per reaction. As total L-DNA copy number decreased, both fluorophore-quencher signal (green) and intercalator signal (red) decreased in magnitude. End-labeled strands retained *t*_m_ identification even when strand copy count decreased. Dashed lines indicate L-DNA *t*_m_ measured by a fluorophore-quencher signal.

The second key design challenge for LHRM was determining how to discriminate between double-stranded L-DNA and D-DNA melting behavior in a single sample. Apparently, all readily available intercalating dyes do not discriminate between enantiomeric DNA (see Supporting Information, Table S9). Therefore, if double-stranded L-DNA and double-stranded D-DNA are combined in a single reaction, the intercalation melt signal reports a composite melt curve. To overcome this L-DNA and D-DNA intercalating crosstalk, L-DNA was end-labeled with Texas Red. The goal was to measure only double-stranded DNA PCR product fluorescence signal on the green optical channel using LCGreen intercalating dye and measure only double-stranded L-DNA fluorescence signal on the orange optical channel using Texas Red fluorophore and quencher end-labeling. Figure 6A demonstrates intercalating crosstalk (i.e., detectable melt signals on both fluorophore-quencher and intercalator channels) when samples contained 4 × 10^11^ copies of double-stranded L-DNA. Samples with 1 × 10^11^ copies of double-stranded L-DNA (Figure 6C) produced sufficient fluorophore-quencher signal for accurate L-DNA melt measurements with little crosstalk contribution detectable in the intercalator channel, and therefore, this L-DNA copy count was selected for further LHRM development.

**Figure 7:**
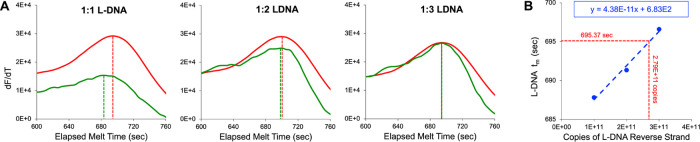
(A) Representative wild-type D-DNA polymerase chain reaction (PCR) product (red) and internal comparator L-DNA (green) derivative melt plots analyzed by LHRM analysis containing double-stranded L-DNA at 1:1, 1:2, and 1:3 ratios of forward to reverse L-DNA strands with 1 × 10^11^ forward strand copies and 1 × 10^11^, 2 × 10^11^, and 3 × 10^11^ reverse strand copies per reaction, respectively. Dashed lines indicate D-DNA PCR product *t*_m_ (red) and L-DNA *t*_m_ (green). (B) There is a positive linear relationship (dashed blue line) between number of L-DNA reverse strand copies and L-DNA *t*_m_ measured via fluorophore-quencher signal. This relationship suggested that an L-DNA forward to reverse strand ratio of 1:2.79 (1 × 10^11^ forward strand copies and 2.79 × 10^11^ reverse strand copies per reaction) would match drug-susceptible L-DNA *t*_m_ to the drug-susceptible D-DNA PCR product average *t*_m_ of 695 s (dashed red lines).

The third design challenge was matching the melt characteristics of the L-DNA melt comparator to the melt characteristics of the wild-type PCR product to support the initial melt matching assumption. Several factors made matching the melt characteristics of the two difficult. It is well-known that the L-DNA sequence end-labeling used to overcome single tube detection also changes the DNA’s melt temperature, even with a 5-base spacer on the quencher strand.^49^ Previous reports have also established that total DNA concentration and strand ratio affect the melt temperature.^50−52^ In addition to these well-known factors, even when they have the same sequence, for unknown reasons, unlabeled double-stranded L-DNA and D-DNA have a small difference in melt temperatures measured by intercalation (Table S9). Since the strand ratio was the easiest to adjust, the strand ratio of the added L-DNA was empirically modified to compensate for these other factors and ultimately match the L-DNA and D-DNA wild-type melt characteristics.^33^ The theory behind this report’s experimental tuning strategy is detailed in a complementary work by Spurlock et al., in 2024.^23^ Spurlock et al., in 2024, detailed the theory behind the effects of DNA concentration and strand ratio on annealing,^23^ while this report experimentally demonstrates its use in melt analysis. As Figure 7A demonstrates, different L-DNA forward to reverse strand ratios shift L-DNA *t*_m_ (Table S8). This phenomenon was used to compensate for all factors discussed above and achieve an empirical melt match between drug-susceptible L-DNA and D-DNA (right most panel in Figure 7A). As reverse strand L-DNA copy count increased, L-DNA *t*_m_ increased (Figure 7A and Table S8). A positive linear relationship between the number of L-DNA reverse strand copies per reaction and L-DNA *t*_m_ indicated that 2.79 × 10^11^ L-DNA reverse strand copies (and 1 × 10^11^ L-DNA forward strand copies) per reaction would produce the 695 s *t*_m_ matching average wild-type PCR product *t*_m_ (Figure 7B). The optimal L-DNA strand ratio was rounded up from 1:2.79 to 1:3 (1 × 10^11^ L-DNA forward strand copies and 3 × 10^11^ L-DNA reverse strand copies per reaction) for ease of sample preparation in LHRM. This method produced an average melt difference of approximately 1 s between drug-susceptible L-DNA and wild-type PCR product (top row in Table S6). In further support of melt matching, there was no statistical difference between *t*_m_’s of drug-susceptible L-DNA and wild-type PCR product (*p* > 0.05, paired *t* test, *n* = 3 trials in triplicate). It is important to note that although this study sought to match the L-DNA melt and PCR product melt, it is not critical to do so. Even without tuning L-DNA to make the melt difference zero, for a fixed concentration of L-DNA in every sample, the *t*_m_ difference will still be a constant in the system and samples can be classified as drug-susceptible when sample *t*_m_ difference equals that constant. Alternatively, intentionally tuning for an excessively large melt mismatch between L-DNA and the wild-type PCR product may help to minimize intercalating crosstalk.

L-DNA tuning and time-scale melt analysis contribute to the simplicity of LHRM single sample classification, i.e., a sample is susceptible if its *t*_m_ difference equals zero. A given LHRM sample’s *t*_m_ difference can only consist of discrete values in multiples of 5.27 s. Discrete melt data every 5.27 s enable easier LHRM classification as melt data are inherently grouped into distinct time points and will clearly have *t*_m_ differences of zero or not (Figure 5). The discrete nature of LHRM melt data is an artifact of the instrument melting ramp rate of 0.025 °C/s and the fluorescence sampling rate of one acquisition per 5.27 s. In contrast to discrete LHRM melt data, sample *T*_m_’s resulting from standard HRM analysis are continuous in nature. As a result, standard HRM analysis produces a spread of data points with less separation between sample types (Figures 2 and 3). Unlike LHRM’s discrete melt data, this continuous spread of data requires grouping into subsets using multiple samples to form the drug-susceptible classification cutoffs.

In this initial LHRM report, a QuantStudio 5 was used to show that LHRM can provide similar performance to standard HRM with use of a single sample. It is important to note, however, that the instrument itself is not critical for the conclusions of this work. Although not confirmed in this report, some features of LHRM suggest this strategy would work with less capable instrument designs. For example, LHRM does not use temperature-based melt reporting determined by instrument calibration. Instead, LHRM utilizes time-defined melt analysis. Measurements were quantified using time instead of temperature for two reasons. First, time-quantified melt measurements are based on raw data unaffected by instrument calibration errors present in the *T*_m_.^37^ Second, this strategy facilitates future LHRM measurements in other types of real-time PCR instruments that are not as well-calibrated as the QuantStudio 5 instrument. To use an available real-time PCR instrument, it must at a minimum have melt analysis capabilities built into its software. Although existing real-time PCR instruments commonly have melt analysis capabilities for product assessment purposes, the instruments generally lack the necessary resolution for high sensitivity SNP scanning by melting.^53^ Performing LHRM on a real-time PCR instrument requires access to sample times and fluorescence values recorded during heating of the melt procedure. The resolution of LHRM classification within a single sample is still limited by correct differentiation between an L-DNA comparator sequence and a PCR product sequence that can differ by only a single base. Among the many factors that make this differentiation challenging is the ratio of the fluorescence sampling rate compared to the instrument’s heating rate. A higher ratio makes differentiation easier. In this report, the ratio is 7.59 fluorescent samples collected per °C. Since it is relatively easy to set the instrument’s desired continuous ramp rate, the maximum sampling rate of the instrument is likely the determining factor that limits the resolution of this within-sample method. Future work is required to determine if the L-DNA reagent-based strategy can be implemented on other instruments with real-time PCR and built-in melt analysis data generation capabilities.

## Conclusions

By including L-DNA for reagent-based calibration in every sample, LHRM successfully classifies PCR melt products as INH-susceptible or not based on within-sample differences between an L-DNA comparator and an unknown PCR product. LHRM achieves comparable classification specificity and sensitivity to standard HRM with single sample analysis. With further development, LHRM shows promise as an initial drug susceptibility screen that may be incorporated into the TB clinical treatment algorithm where real-time PCR instruments are available.

## Supporting Information Available

Additional experimental details, methods, and materials, including oligonucleotide sequences for this work; complete details regarding approach, analysis, and statistics of standard HRM and LHRM; standard HRM and LHRM tabulated melt data; experiments exploring heating variability of the QuantStudio 5; comparison of LHRM and standard HRM analysis strategies; alternative classification strategies that establish drug-susceptible cutoff points for standard HRM and LHRM using a maximized Youden J Statistic; experiments exploring sample-to-sample salt variability on standard HRM and LHRM classification; experimental characterization of L-DNA; comparison of equal concentration 1:1 D-DNA and unlabeled L-DNA; and representative PCR amplification curves of samples with and without L-DNA (PDF)
