# Supplementary material for: L-DNA-Based Melt Analysis Enables Within-Sample Validation of PCR Products
Source: Anal Chem. 2024 Jul 8;96(29):11897–905. doi: 10.1021/acs.analchem.4c01611 (PMC11270519; doi:10.1021/acs.analchem.4c01611)
Supplement: Supplementary file 1 — ac4c01611_si_001.pdf [file ac4c01611_si_001.pdf]

Supporting Information

L-DNA-based melt analysis enables within-sample validation of PCR products

Nicole A. Malofsky[1], Dalton J. Nelson[1], Megan E. Pask[1], Frederick R. Haselton[1*]

[1] Department of Biomedical Engineering, Vanderbilt University, Nashville, TN, 37235, United States

* To whom correspondence should be addressed. Tel: (615)-322-6622; Fax: (615)-343-7919; Email: Rick.Haselton@vanderbilt.edu

Table of Contents

Oligonucleotides …… S2
Standard HRM approach, analysis, and statistics …… S3
LHRM approach, analysis, and statistics …… S4-S5
Standard HRM melt data …… S6
Heating variability of the QuantStudio™ 5 …… S7
LHRM melt data …… S8
Comparison of LHRM and standard HRM analysis strategies …… S9
Establishing drug-susceptible classification cutoff points using a maximized Youden J Statistic…… S10-S11
L-DNA overcomes the effects of sample-to-sample salt variability on classification …… S12
L-DNA characterization …… S13
Comparison of equal concentration 1:1 D-DNA and unlabeled 1:1 L-DNA …… S14
PCR results …… S15

Oligonucleotides

Table S1. Oligonucleotide sequences designed for standard HRM and LHRM. DNA is denoted as D-DNA or L-DNA. Single or multi-base changes of interest in mutants are indicated in red.

ID	DNA Type	Description	Amino Acid Change	Nucleotide Base Change	Sequence (5' → 3')
MEP183	D-DNA	Wild-type drug-susceptible target	None	None	AT CTG GTC GGC CCC GAA CCC GAG GCT GCT CCG CTG GAG CAG ATG GGC TTG GGC TGG AAG AGC TCG TAT GGC ACC GGA ACC GGT AAG GAC GCG ATC ACC AGC GGC ATC GAG GTC GTA TGG ACG AAC ACC CCG ACG AAA TGG GAC AAC AGT TTC CTC GAG ATC CTG TAC GGC TAC GAG TGG GAG CTG ACG AAG AGC CCT GCT
MEP184	D-DNA	Mutant target	S315T	G944C	C TCG TAT GGC ACC GGA ACC GGT AAG GAC GCG ATC ACC ACC GGC ATC GAG GTC GTA TGG ACG AAC ACC CCG ACG AAA TGG GAC AAC AGT T
MEP185	D-DNA	Mutant target	S315N	G944A	C TCG TAT GGC ACC GGA ACC GGT AAG GAC GCG ATC ACC AAC GGC ATC GAG GTC GTA TGG ACG AAC ACC CCG ACG AAA TGG GAC AAC AGT T
MEP186	D-DNA	Mutant target	S315I	G944T	C TCG TAT GGC ACC GGA ACC GGT AAG GAC GCG ATC ACC ATC GGC ATC GAG GTC GTA TGG ACG AAC ACC CCG ACG AAA TGG GAC AAC AGT T
MEP187	D-DNA	Mutant target	S315R	C945A	C TCG TAT GGC ACC GGA ACC GGT AAG GAC GCG ATC ACC AGA GGC ATC GAG GTC GTA TGG ACG AAC ACC CCG ACG AAA TGG GAC AAC AGT T
MEP188	D-DNA	Mutant target	S315G	A943G	C TCG TAT GGC ACC GGA ACC GGT AAG GAC GCG ATC ACC GGC GGC ATC GAG GTC GTA TGG ACG AAC ACC CCG ACG AAA TGG GAC AAC AGT T
MEP189	D-DNA	Mutant target	S315L	A943C + G944T	C TCG TAT GGC ACC GGA ACC GGT AAG GAC GCG ATC ACC CTC GGC ATC GAG GTC GTA TGG ACG AAC ACC CCG ACG AAA TGG GAC AAC AGT T
MEP197	D-DNA	Mutant target	S315T + A312V	G944C + C935T	C ACC GGA ACC GGT AAG GAC GTG ATC ACC ACC GGC ATC GAG GTC GTA TGG ACG AAC A
MEP198	D-DNA	Mutant target	S315T + G316D	G944C + G947A	C ACC GGA ACC GGT AAG GAC GCG ATC ACC ACC GAC ATC GAG GTC GTA TGG ACG AAC A
MEP199	D-DNA	Mutant target	S315T + G316D + A312V	G944C + G947A + C935T	C ACC GGA ACC GGT AAG GAC GTG ATC ACC ACC GAC ATC GAG GTC GTA TGG ACG AAC A
23FEB_katGf56_TXR	L-DNA	Wild-type drug-susceptible comparator forward strand with end-labeling	None	None	/TXR/ C ACC GGA ACC GGT AAG GAC GCG ATC ACC AGC GGC ATC GAG GTC GTA TGG ACG AAC A
23FEB_katG_56_Rcmp+5_BHQ2	L-DNA	Wild-type drug-susceptible comparator reverse strand with end-labeling	None	None	T GTT CGT CCA TAC GAC CTC GAT GCC GCT GGT GAT CGC GTC CTT ACC GGT TCC GGT GCC ATA /BHQ2/
MEP176	D-DNA	56 bp PCR amplicon forward primer	N/A	N/A	CAC CGG AAC CGG TAA GG
MEP177	D-DNA	56 bp PCR amplicon reverse primer	N/A	N/A	TGT TCG TCC ATA CGA CCT C

Standard HRM approach, statistics, and analysis

Approach: The standard HRM approach for drug susceptibility screening is based on a two-sample comparison of T_m's between an unknown PCR product and a known drug-susceptible PCR product (**Figure S1**). Reactions were performed in the Applied Biosystems™ QuantStudio™ 5 real-time PCR thermal cycler (Thermo Fisher Scientific #A28137). This highly capable instrument was selected to facilitate standard HRM performance as a state-of-the-art comparison method for LHRM. QuantStudio™ 5 uses a 96-well format[1] (Applied Biosystems™ #4483485). Reactions had a 20 μL final volume containing 1X of SensiFAST™ Probe No-ROX Kit (Bioline #BIO-86005), 1X LCGreen® Plus (BioFire® Defense, LLC #BCHM-ASY-005), and 250 nM of each *katG*-specific primer (MEP176 and MEP177). Each target sample contained a final concentration of wild-type (MEP183) or mutant (MEP184-189,197-199) single-stranded DNA target at 2×10^6 copies per reaction. An example of a standard HRM reaction setup is outlined in **Table S2**. Samples were loaded into the 96-well plate such that each set of sample type triplicates were loaded into consecutive wells in the same row, except for experiments testing heating variability across the 96-well plate in which wild-type and S315T samples were loaded into mirrored quadrants of the 96-well plate. PCR reactions were initiated with a 95 °C hold for 2 min followed by 40 cycles of 95 ºC for 5 sec and 59 ºC for 20 sec. Fluorescence was measured at the end of the annealing/extension step (59 ºC). A high resolution melt was performed immediately following PCR by annealing 95 ºC to 50 ºC at 0.1 ºC/sec followed by melting 65 ºC to 95 ºC at 0.025 ºC/sec (continuous acquisition mode). This melting ramp rate is often used in QuantStudio™ 5 HRM mutation scanning[2–5]. Double-stranded DNA PCR product fluorescence was monitored during PCR and during the melt reaction using LCGreen® Plus on the green optical channel (excitation 470±15 / emission 520±15). The QuantStudio™ 5 was initially factory-calibrated for optical and thermal accuracy[6]. All standard calibration statuses (ROI/Uniformity, Background, Dyes)[6] were kept current. Custom dye calibration and custom melt curve calibration[6] were performed for LCGreen® Plus.

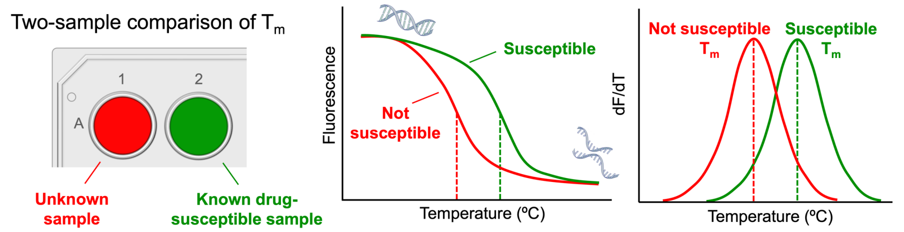

Figure S1. Standard HRM relies on comparison of temperature-based melt characteristics between two samples. Dashed lines indicate T_m's of drug-susceptible (green) and not drug-susceptible (red) PCR products.

Table S2. Example reaction setup without L-DNA additive.

Component	[Stock]	[Final]	Volume (μL)
Nuclease-free water	-	-	5
SensiFAST™ Probe No-ROX Kit	2x	1x	10
LCGreen® Plus	10x	1x	2
Forward primer	10 μM	250 nM	0.5
Reverse primer	10 μM	250 nM	0.5
Target	-	-	2
		Total Volume	20

Analysis and statistics: PCR quantification cycle (C_q) was determined with the QuantStudio™ 5 Design and Analysis Software. Non-amplifying samples did not report C_q and were excluded from the data analysis. Amplifying samples with C_q over 35 were excluded from the data analysis because they did not achieve the PCR plateau phase. Representative PCR amplification curves of samples are included in **Figure S11**. T_m was calculated with the proprietary QuantStudio™ 5 Design and Analysis Software based on the first derivative of fluorescence with respect to temperature. Based on T_m analysis of all samples, T_m cutoff points were established to maximize test specificity when classifying standard HRM analyzed samples as drug-susceptible or not. Specificity was maximized to decrease the false positive rate, i.e., decrease the misdiagnosis of variant samples as drug-susceptible. This maximized specificity strategy is often used for HRM classification of TB samples with drug resistance[7–9]. Each test sample was individually classified. A sample was classified as drug-susceptible when PCR product T_m was within the drug-susceptible T_m cutoff range of 82.4 ºC and 82.5 ºC. Since true positives are known, standard HRM was assessed for its sensitivity and specificity using this T_m cutoff range to classify drug susceptibility among 9 true drug-susceptible samples (n=3 trials of wild-type in triplicate) and 81 true not drug-susceptible samples (n=3 trials of 9 variant types in triplicate). The true positive (sensitivity) rate was calculated as the percentage of drug-susceptible (+) test results out of all true wild-type (+) samples. The true negative (specificity) rate was calculated as the percentage of not drug-susceptible (-) test results out of all true variant (-) samples. In the experiment testing heating variability, significance was evaluated using T_m comparison (unpaired *t* test, significance level of α=0.95) of 96-well plate quadrants of S315T as compared to wild-type (n=1 trial with 24 replicates per sample type). All statistics were performed in Microsoft® Excel 2022 except for the sensitivity and specificity analysis that was performed in Python.

LHRM approach, statistics, and analysis

Approach: LHRM for drug susceptibility screening is based on elapsed melt time (t_m) comparison between an unknown PCR product and a drug-susceptible L-DNA comparator within a single sample (**Figure S2**). To ensure a fair comparison between LHRM and standard HRM, both methods were tested using the same QuantStudio™ 5 instrument. LHRM used identical PCR cycling, PCR fluorescence monitoring, PCR quantification, melt reaction cycling, reaction loading placement, and heating variability test setup as standard HRM. LHRM statistics were identical to that of standard HRM, except for a data subset testing heating variability. Key changes from standard HRM are the inclusion of an additional reagent (L-DNA), monitoring melt reaction fluorescence on a second optical channel, and analysis of fluorescence changes as a function of time from the start of the QuantStudio™ 5 continuous mode melt instead of melt temperature provided by the instrument's calibration.

A double-stranded L-DNA drug-susceptible comparator was synthesized using left-helical enantiomeric DNA bases (i.e., L-DNA) [10] with an identical sequence to the known drug-susceptible *katG* sequence. The 56-base L-DNA was synthesized with the same length and sequence as the drug-susceptible PCR amplicon. The double-stranded L-DNA was end-labeled with Texas Red (TXR) fluorophore and Black Hole Quencher 2 (BHQ2) quencher to monitor its behavior during melting on the orange fluorescence channel (excitation 580±10 / emission 623±14). L-DNA fluorescence signal was scaled up by a factor of 18 in derivative melt plots for visual comparison with the PCR product's higher fluorescence signal. Detailed information on the L-DNA oligonucleotide sequences used in these studies are shown in **Table S1**. LHRM reactions included 2 μL of L-DNA mix with final copy counts of 1×10^{11} copies TXR-labeled forward strand L-DNA (23FEB_katGf56_TXR) and 3×10^{11} copies BHQ2-labeled reverse strand L-DNA (23FEB_katG_56_Rcmp+5_BHQ2) per reaction. An example reaction setup containing the L-DNA additive is outlined in **Table S3**.

To ensure identical melt characteristics of D-DNA and end-labeled L-DNA, additional experiments were performed varying L-DNA strand concentration and strand ratio. In experiments varying L-DNA strand concentrations, reaction component deviations included 100 nM final concentration of each *katG*-specific primer and 1×10^{11}, 2×10^{11}, and 4×10^{11} copies of L-DNA strands (forward and reverse) per reaction. In experiments varying L-DNA forward to reverse strand ratio, reaction component deviations included 100 nM final concentration of each *katG*-specific primer and 2 μL of L-DNA mix at 1:1, 1:2, and 1:3 ratios of forward to reverse strands for final L-DNA copy numbers of 1×10^{11} copies of forward strand plus 1×10^{11}, 2×10^{11}, and 3×10^{11} copies of reverse strand, respectively. Linear interpolation of three different L-DNA strand ratios was used to determine the relationship between copies of L-DNA reverse strands per reaction and L-DNA melt measurement. The L-DNA reverse strand copy number with a melt measurement matching that of wild-type PCR product was selected.

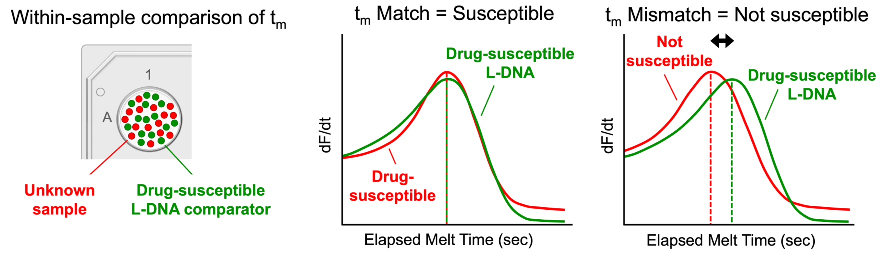

Figure S2. LHRM compares time-based melt characteristics within one sample using a drug-susceptible L-DNA internal comparator. Dashed lines indicate PCR product t_m (red) and drug-susceptible L-DNA t_m (green).

Table S3. Example reaction setup containing L-DNA additive.

Component	[Stock]	[Final]	Volume (μL)
Nuclease-free water	-	-	3
SensiFAST™ Probe No-ROX Kit	2x	1x	10
LCGreen® Plus	10x	1x	2
Forward primer	10 μM	250 nM	0.5
Reverse primer	10 μM	250 nM	0.5
L-DNA mix	-	-	2
Target	-	-	2
		Total Volume	20

Analysis and statistics: Representative PCR amplification curves of samples containing L-DNA are included in **Figure S12**. Elapsed melt time (t_m) was calculated from the second degree Savitsky–Golay polynomials[11] at each point (performed in MATLAB 2023A) based on the first derivative of fluorescence with respect to elapsed melt time. Elapsed melt time is a means of T_m reporting derived from the uncalibrated QuantStudio™ 5 raw data. Here, t_m is defined as the elapsed melt time (in seconds) to reach the maximum derivative of fluorescence with respect to elapsed melt time. Significant differences between wild-type PCR product and L-DNA within each sample were assessed using paired *t* tests (of t_m) with a significance level of $\alpha = 0.95$ (n = 3 trials in triplicate). Test samples were classified as drug-susceptible when sample t_m difference was zero. LHRM classification criteria is based on our assumption that L-DNA and PCR product melt characteristics are identical if and only if their sequences match. Specificity was maximized to decrease the false positive rate, i.e., decrease the misdiagnosis of variant samples as drug susceptible. Since true positives are known, LHRM was assessed for its sensitivity and specificity using a t_m difference of zero to classify drug susceptibility among 9 true drug-susceptible samples (n=3 trials of wild-type in triplicate) and 79 true not drug-susceptible samples (n=3 trials of 9 variant types in triplicate, except variant S315T+G316D+A312V which had one trial with a single replicate due to C_q exclusion).

To directly compare time-based LHRM analysis within a single sample and temperature-based standard HRM analysis between samples, L-DNA-containing samples were also analyzed using standard HRM analysis. Sample T_m was calculated with the proprietary QuantStudio™ 5 Design and Analysis Software. Based on T_m analysis of all samples, T_m cutoff points were established to maximize test specificity when classifying each test sample as drug-susceptible or not. Specificity was maximized to decrease the false positive rate. A sample was classified as drug-susceptible when PCR product T_m was within the drug-susceptible T_m cutoff range of 82.4 ºC and 82.5 ºC. Since true positives are known, classification sensitivity and specificity were assessed using this T_m cutoff range to classify drug susceptibility among 9 true drug-susceptible samples (n=3 trials of wild-type in triplicate) and 79 true not drug-susceptible samples (n=3 trials of 9 variant types in triplicate, except variant S315T+G316D+A312V of one trial with a single replicate due to C_q exclusion).

Alternative strategies exist to establish drug-susceptible classification cutoff points for HRM analysis, and this was explored in *Supporting Information* (see pages **S6-S7**). This supplemental work used a maximized Youden J Statistic[12] to establish drug-susceptible classification cutoff points for the same data sets across standard HRM and LHRM analysis strategies (see pages **S6-S7**). This alternative cutoff strategy generally improved sensitivity and decreased specificity.

In the experiment testing heating variability, significance was evaluated using melt measurement comparison (Mann-Whitney U test, significance level of α= 0.95) of 96-well plate quadrants of S315T as compared to wild-type (n=1 trial with 24 replicates). The heating variability Mann-Whitney U test was performed twice, once using T_m as the melt measurement and once using t_m difference as the melt measurement. All statistics were performed in Microsoft® Excel 2022 except for the sensitivity and specificity analysis that was performed in Python.

Standard HRM melt data

Table S4. Summary of standard HRM T_m's and T_m differences of *katG* wild-type and nine *katG* variants (n=3 trials in triplicate per sample type).

katG **Sample Type**	**Nucleotide Base Change**	**Average PCR Product T_m (ºC±SD)**	**Average T_m Difference from Global Average Known Wild-Type (ºC±SD)**
**Wild-Type**	**None**	**82.47±0.06**	**0.00±0.06**
**S315T**	**G944C**	**82.29±0.13**	**-0.18±0.13**
**S315N**	**G944A**	**81.75±0.03**	**-0.70±0.03**
**S315I**	**G944T**	**81.71±0.08**	**-0.75±0.08**
**S315R**	**C945A**	**81.58±0.08**	**-0.88±0.08**
**S315G**	**A943G**	**83.06±0.14**	**0.61±0.14**
**S315L**	**A943C + G944T**	**82.12±0.03**	**-0.33±0.03**
**S315T + A312V**	**G944C + C935T**	**81.43±0.07**	**-1.02±0.07**
**S315T + G316D**	**G944C + G947A**	**81.64±0.08**	**-0.81±0.08**
**S315T + G316D + A312V**	**G944C + G947A + C935T**	**80.63±0.14**	**-1.82±0.14**

Table S5. Summary of PCR product T_m's and T_m differences of samples containing an L-DNA comparator in every sample and analyzed by standard HRM across wild-type and nine variants (n=3 trials in triplicate per sample type, except variant S315T+G316D+A312V of one trial with a single replicate due to C_q exclusion).

katG **Sample Type**	**Nucleotide Base Change**	**Average PCR Product T_m (ºC±SD)**	**Average T_m Difference from Global Average Known Wild-Type (ºC±SD)**
**Wild-Type**	**None**	**82.51±0.07**	**0.00±0.07**
**S315T**	**G944C**	**82.33±0.12**	**-0.18±0.12**
**S315N**	**G944A**	**81.80±0.03**	**-0.72±0.03**
**S315I**	**G944T**	**81.80±0.03**	**-0.71±0.03**
**S315R**	**C945A**	**81.63±0.08**	**-0.89±0.08**
**S315G**	**A943G**	**83.06±0.15**	**0.55±0.15**
**S315L**	**A943C + G944T**	**82.18±0.04**	**-0.33±0.04**
**S315T + A312V**	**G944C + C935T**	**81.44±0.04**	**-1.07±0.04**
**S315T + G316D**	**G944C + G947A**	**81.70±0.07**	**-0.81±0.07**
**S315T + G316D + A312V**	**G944C + G947A + C935T**	**80.72±0.15**	**-1.79±0.15**

Heating variability of the QuantStudio™ 5

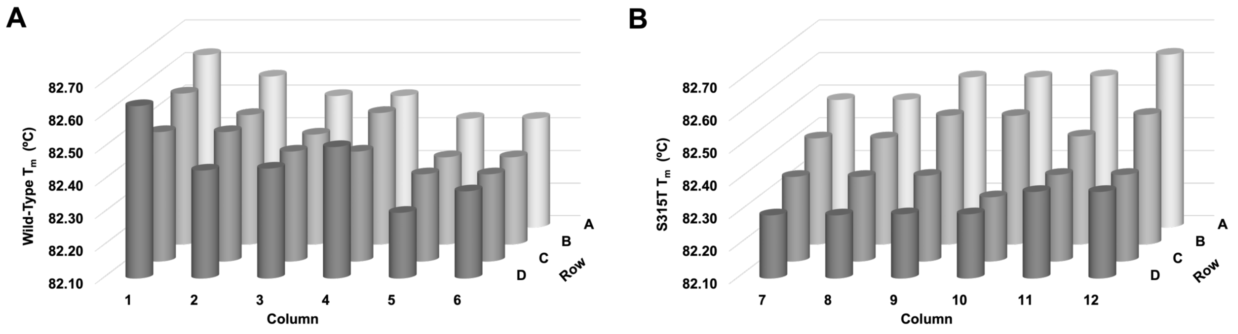

Figure S3. Standard HRM PCR product T_m's of (**A**) wild-type *katG* and (**B**) S315T samples across the top left and top right quadrants, respectively, of a 96-well plate.

LHRM melt data

Table S6. Summary of LHRM t_m's and t_m differences of wild-type and nine variants (n=3 trials in triplicate per sample type, except variant S315T+G316D+A312V which had one trial with a single replicate due to C_q exclusion).

katG Sample Type	Nucleotide Base Change	Average PCR Product t_m (sec±SD)	Average L-DNA t_m (sec±SD)	Average t_m Difference from L-DNA Within Well (sec±SD)
Wild-Type	None	700.79±1.77	701.96±2.63	-1.17±2.32
S315T	G944C	692.60±3.84	700.20±5.24	-7.60±3.82
S315N	G944A	673.30±1.74	699.03±4.42	-25.74±4.11
S315I	G944T	671.54±2.80	699.03±2.34	-27.49±3.51
S315R	C945A	664.52±2.34	700.20±2.60	-35.68±3.51
S315G	A943G	722.43±5.15	700.79±4.10	21.64±4.12
S315L	A943C + G944T	689.09±1.74	699.62±3.15	-10.53±3.72
S315T + A312V	G944C + C935T	657.50±1.79	702.54±3.83	-45.04±3.82
S315T + G316D	G944C + G947A	667.45±3.52	701.96±4.56	-34.51±4.64
S315T + G316D + A312V	G944C + G947A + C935T	634.37±0.09	702.81±3.82	-70.20±8.05

Table S7. Summary of melt characteristics for 24 identical wild-type samples containing L-DNA and 24 identical S315T samples containing L-DNA in the top left and top right quadrants, respectively, of 96-well heating block (n=1 trial). Samples were analyzed by standard HRM analysis for PCR product T_m's and analyzed by LHRM analysis for PCR product t_m's and t_m differences. L-DNA dynamically calibrated to heating variability, enabling wild-type and S315T samples to be successfully differentiated using within-sample t_m differences.

katG Sample Type	Average PCR Product T_m Across All Wells (ºC±SD)	Average PCR Product t_m Across All Wells (sec±SD)	Average t_m Difference (from L-DNA Within Well) Across All Wells (sec±SD)
Wild-Type	82.45±0.09	697.90±3.79	0.66±4.50
S315T	82.43±0.10	697.23±3.81	-4.62±3.24

Comparison of LHRM and standard HRM analysis strategies

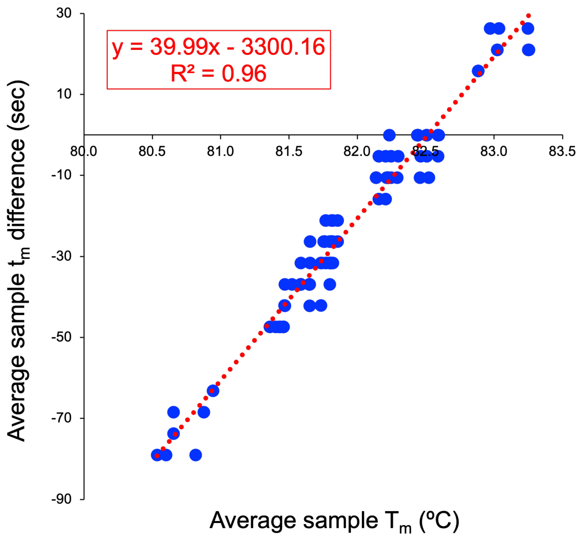

Figure S4. Direct linear relationship (dashed red line) between LHRM analysis (t_m difference) and standard HRM analysis (T_m) of samples containing an L-DNA comparator in every sample across wild-type and nine variant sample types. Each point represents an individual test sample analyzed using two different analysis strategies.

Establishing drug-susceptible classification cutoff points using a maximized Youden J Statistic

In this report, the standard HRM analysis strategy established drug-susceptible classification cutoff points to maximize specificity when classifying samples as drug-susceptible or not. In the context of drug-susceptibility testing, specificity was maximized to decrease the misdiagnosis of variant samples as drug-susceptible. Unlike standard HRM, LHRM drug-susceptible classification criteria were established without relying on analysis of data from multiple samples. LHRM classified samples as drug-susceptible when a sample's t_m difference was zero.

Alternative strategies exist to establish drug-susceptible classification cutoff points for HRM analysis. In this supplemental analysis, a maximized Youden J Statistic[12] was used to establish the drug-susceptible classification cutoff points for both standard HRM and LHRM analysis strategies. By maximizing the Youden J Statistic, which is symmetric in sensitivity and specificity, equal weight is given to false positives and false negatives[12]. This "best overall" approach has trade-offs in the context of drug-susceptibility testing as it prioritizes equally the correct classification of true drug-susceptible cases and true not drug-susceptible cases. The Youden J Statistic requires data from multiple samples to inform classification of a single sample, whether applied to standard HRM or LHRM analysis. Across data sets analyzed by either analysis strategy, each test sample was individually classified. True positive (sensitivity) rate was calculated as the percentage of drug-susceptible (+) test results out of all true wild-type (+) samples. The true negative (specificity) rate was calculated as the percentage of not drug-susceptible (-) test results out of all true variant (-) samples. All Sensitivity and specificity analysis was performed in Python.

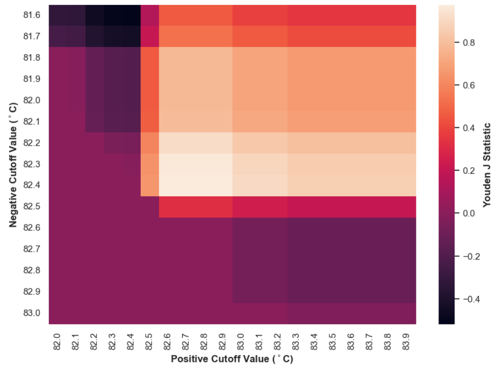

Figure S5. Heatmap of the Youden J Statistic across upper (positive) and lower (negative) bound drug-susceptible T_m cutoff values (in ºC) from samples without L-DNA and analyzed using standard HRM analysis.

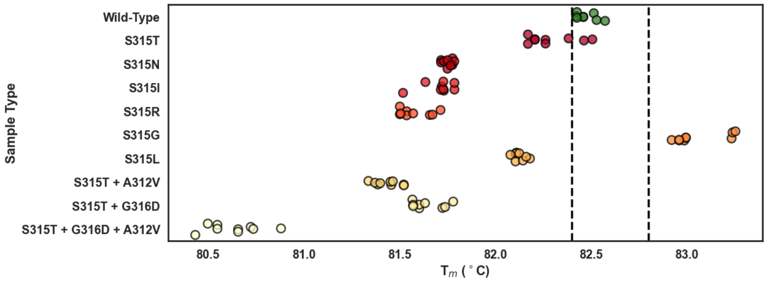

Figure S6. PCR product melt temperatures of samples without L-DNA and analyzed by standard HRM across wild-type (green) and nine variants (n=3 trials in triplicate per sample type). Samples were classified as drug-susceptible or not by comparing sample T_m to the drug-susceptible T_m cutoff range of 82.4 ºC and 82.8 ºC (indicated by dashed black lines). Each point represents an individual test sample.

The Youden J Statistic was utilized to determine the sensitivity and specificity of samples without L-DNA and analyzed by standard HRM analysis. The Youden J Statistic was calculated for upper and lower bound drug-susceptible T_m cutoff values (in ºC) and plotted as a heatmap (**Figure S5**). The maximized Youden J Statistic in the heatmap established T_m cutoff values for standard HRM sample classification as drug-susceptible or not. This strategy is often used for HRM classification of TB samples with drug-resistance[7–9]. A sample was classified as drug-susceptible when PCR product T_m was within the drug-susceptible T_m cutoff range of 82.4 ºC and 82.8 ºC. Since true positives are known, the sample set was assessed for sensitivity and specificity using this T_m cutoff range when classifying drug-susceptibility among 9 true drug-susceptible samples (n=3 trials of wild-type in triplicate) and 81 true not drug-susceptible samples (n=3 trials of 9 variant types in triplicate). Using a maximized Youden J Statistic, samples analyzed by standard HRM performed at 100% sensitivity and 97.5% specificity. As compared to standard HRM metrics produced using a maximized specificity in this report, Youden-based metrics increased sensitivity by 33.3% but decreased specificity by 1.3%. Youden-based sample classification accuracy is illustrated in **Figure S6**. In particular, standard HRM misclassification is illustrated by two S315T samples within the drug-susceptible cutoff range (**Figure S6**).

The Youden J Statistic was utilized to determine the sensitivity and specificity of samples containing L-DNA but analyzed by standard HRM analysis. The Youden J Statistic was calculated for upper and lower bound drug-susceptible cutoff values and plotted as a heatmap (**Figure S7**). The maximized Youden J Statistic in the heatmap established T_m cutoff values for sample classification as drug-susceptible or not. A sample was classified as drug-susceptible when sample T_m was within the drug-susceptible T_m cutoff range of 82.4ºC to 82.8ºC. Notably, this Youden-based cutoff range was the same for standard HRM analysis of samples with L-DNA (**Figure S8**) and without L-DNA (**Figure S6**). Since true positives are known, the sample set was assessed for sensitivity and specificity using this T_m cutoff range when classifying drug-susceptibility among 9 true drug-susceptible samples (n=3 trials of wild-type in triplicate) and 79 true not drug-susceptible samples (n=3 trials of 9 variant types in triplicate, except variant S315T+G316D+A312V of one trial with a single replicate due to C_q exclusion). Using a maximized Youden J Statistic, L-DNA-containing samples analyzed by standard HRM performed at 100% sensitivity and 96.2% specificity. As compared to standard HRM metrics produced using a maximized

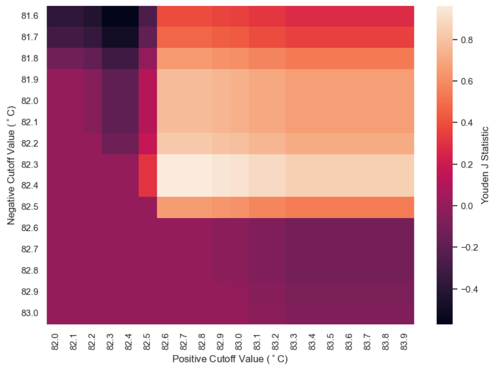

Figure S7. Heatmap of the Youden J Statistic across upper (positive) and lower (negative) bound drug-susceptible T_m cutoff values (in ºC) from samples containing L-DNA but analyzed using standard HRM analysis.

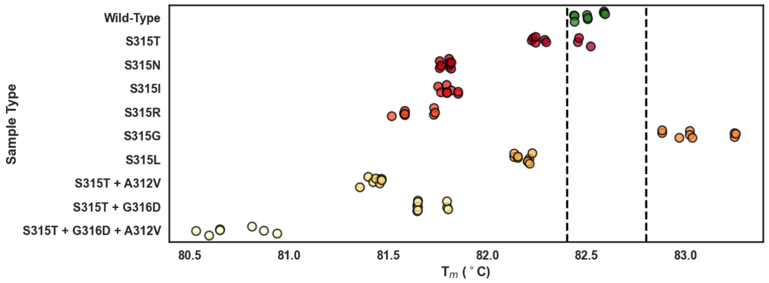

Figure S8. PCR product melt temperatures of samples containing L-DNA but analyzed by standard HRM analysis, including wild-type (green) and nine variants (n=3 trials in triplicate per sample type, except variant S315T+G316D+A312V of one trial with a single replicate due to C_q exclusion). Samples were classified as drug-susceptible or not by comparing sample T_m to the drug-susceptible T_m cutoff range of 82.4ºC to 82.8ºC (indicated by dashed black lines). Each point represents an individual test sample.

specificity on L-DNA-containing samples in this report, Youden-based metrics increased sensitivity by 66.67% but decreased specificity by 1.3%. Youden-based sample classification accuracy is illustrated in **Figure S8**. In particular, standard HRM misclassification is illustrated by three S315T samples within the drug-susceptible cutoff range (**Figure S8**).

The Youden J Statistic was also utilized to determine the sensitivity and specificity of samples containing L-DNA and analyzed by LHRM analysis. The Youden J Statistic was calculated for upper and lower bound drug-susceptible cutoff values and plotted as a heatmap (**Figure S9**). The maximized Youden J Statistic in the heatmap established t_m difference cutoff values for LHRM sample classification as drug-susceptible or not. A sample was classified as drug-susceptible when sample t_m difference was within the drug-susceptible t_m difference cutoff range of -8 sec to 8 sec. Since true positives are known, LHRM was assessed for its sensitivity and specificity using this t_m difference cutoff range when classifying drug-susceptibility among 9 true drug-susceptible samples (n=3 trials of wild-type in triplicate) and 79 true not drug-susceptible samples (n=3 trials of 9 variant types in triplicate, except variant S315T+G316D+A312V of one trial with a single replicate due to C_q exclusion). Using a maximized Youden J Statistic, LHRM performed at 100% sensitivity and 92.4% specificity. As compared to LHRM metrics produced using a maximized specificity on L-DNA-containing samples in this report, Youden-based metrics increased sensitivity by 22.2% but decreased specificity by 6.3%. Youden-based sample classification accuracy is illustrated in **Figure S10**. In particular, LHRM misclassification is illustrated by six S315T samples within the drug-susceptible cutoff range (**Figure S10**). This increase in false positive rate (i.e., misdiagnosis of not drug-susceptible cases) would be detrimental in the context of INH drug-susceptibility testing because those patients would remain on ineffective TB treatment by INH.

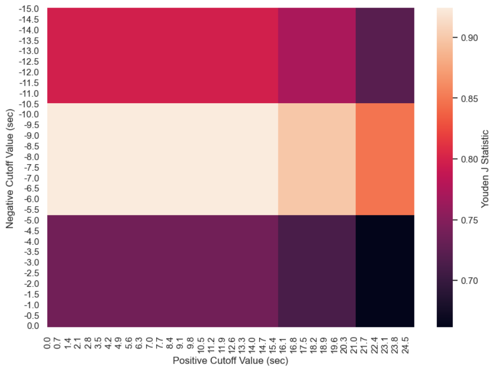

Figure S9. Heatmap of the Youden J Statistic across upper (positive) and lower (negative) bound drug-susceptible t_m difference cutoff values (in seconds) from samples containing L-DNA and analyzed using LHRM analysis.

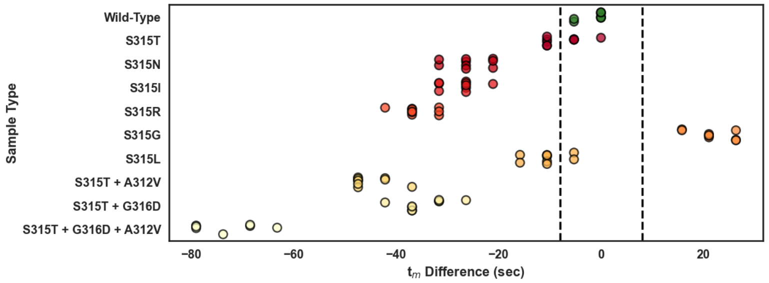

Figure S10. Within-sample t_m differences of samples containing L-DNA and analyzed by LHRM analysis, including wild-type (green) and nine variants (n=3 trials in triplicate per sample type, except variant S315T+G316D+A312V of one trial with a single replicate due to C_q exclusion). Samples were classified as drug-susceptible or not by comparing sample t_m difference to the drug-susceptible t_m difference cutoff range of -8 sec to 8 sec (indicated by dashed black lines). Each point represents an individual test sample.

L-DNA overcomes the effects of sample-to-sample salt variability on classification

Instrument-based heating errors change the hybridization of a sample so this supplemental, proof-of-concept study tested whether other factors affecting hybridization could also be overcome using LHRM. While no other methodological errors were identified in HRM testing, it was speculated that some types of sample preparation errors could introduce systematic hybridization changes which could also be corrected using L-DNA. Possible errors may include culture media carryover, extraction errors, kit-to-kit master mix differences, or sample-to-sample salt concentration variability resulting from reagent pipetting errors.[13] Of these, differences in salt concentration (which are known to alter DNA melting behavior and melting temperature[14]) were the easiest to test. Using standard HRM, salt additions produced statistically significant changes in wild-type sample T_m as compared to standard preparation samples ($p<0.05$, Wilcoxon signed-rank test, 36 replicates per sample type) and would ultimately cause wild-type sample misclassification. Average PCR product T_m (mean±SD) was 82.49±0.10 ºC for standard preparation wild-type samples and 83.18±0.10 ºC for salt-additive wild-type samples. This suggests that preparation errors altering salt concentrations would not only likely misclassify wild-type samples but also likely misclassify variants by masking small mutation-induced melt changes.

Sample-to-sample preparation errors were further evaluated to determine if LHRM could overcome salt hybridization effects. LHRM reactions were first assessed using PCR product melt measurement alone for direct comparison to standard HRM reactions that did not contain L-DNA. Consistent with the previous results, standard melt analysis (using PCR product melt measurements alone) for LHRM reactions could not correctly characterize salt-additive wild-type samples as drug-susceptible. Specifically, there was a significant difference between salt-additive and standard preparation wild-type reactions when PCR product t_m was used alone ($p<0.05$, Wilcoxon signed-rank test). Average PCR product t_m (mean±SD) was 699.53±4.06 sec for standard preparation wild-type samples and 727.59±3.44 sec for salt-additive wild-type samples. However, as seen with heating variability, including a fixed amount of L-DNA in each sample provided a consistent comparator hybridization event that was used to reduce sample-to-sample sample preparation variability affecting hybridization. Using the L-DNA to PCR product melt difference, wild-type samples were correctly characterized as drug-susceptible whether or not they contained excess sodium. Specifically, wild-type t_m differences were not significantly different when salt concentration increased ($p>0.05$, Wilcoxon signed-rank test, 36 replicates per sample type). Average t_m difference (mean±SD) was 1.02±3.94 sec for standard preparation wild-type samples and 3.07±3.17 sec for salt-additive wild-type samples. As hypothesized, LHRM corrected for error-induced melt shifts by including L-DNA in every sample, and thus overcame hybridization effects of sample preparation and heating variability. This data was consistent with historical evidence that L-DNA has identical conformation transitions in the presence of salts as their D-DNA counterparts[15].

Experiments testing the effect of sample-to-sample salt concentration variability were performed in the Applied Biosystems™ QuantStudio™ 5 real-time PCR thermal cycler (Thermo Fisher Scientific #A28137). Reactions had a 20 μL final volume containing 1X of SensiFAST™ Probe No-ROX Kit (Bioline #BIO-86005), 1X LCGreen® Plus (BioFire® Defense, LLC #BCHM-ASY-005), and 250 nM of each *katG*-specific primer (MEP176 and MEP177). Each target sample contained a final concentration of wild-type (MEP183) single-stranded DNA target at 2×10^6 copies per reaction. Salt-additive samples included 35 mM sodium chloride (Sigma Aldrich #S5150-1L) per reaction to simulate viral transport media equivalent salt contributions from possible extraction error. LHRM reactions included 2 μL of L-DNA mix with final copy counts of 1×10^{11} copies TXR-labeled forward strand L-DNA (23FEB_katGf56_TXR) and 3×10^{11} copies BHQ2-labeled reverse strand L-DNA (23FEB_katG_56_Rcmp+5_BHQ2) per reaction.

Salt-additive experiments assumed that thermal characteristics held constant plate-to-plate. Standard preparation and salt-additive wild-type samples were loaded into two plates of matching well-to-well standard preparation versus salt-additive samples. PCR reactions were initiated with a 95 °C hold for 2 min followed by 40 cycles of 95 ºC for 5 sec and 59 ºC for 20 sec. Fluorescence was measured at the end of the annealing/extension step (59 ºC). A high resolution melt was performed immediately following PCR by annealing 95 ºC to 50 ºC at 0.1 ºC/sec followed by melting 65 ºC to 95 ºC at 0.025 ºC/sec. Melt fluorescence was measured using the continuous acquisition mode. Double-stranded DNA PCR product fluorescence was monitored during PCR and during the melt reaction using LCGreen® Plus on the green optical channel (excitation 470±15 / emission 520±15). Double-stranded L-DNA (in LHRM reactions) was monitored during the melt reaction using end-labeling (Texas Red fluorophore and Black Hole Quencher 2 quencher) on the orange fluorescence channel (excitation 580±10 / emission 623±14).

PCR quantification cycle (C_q) was determined with the QuantStudio™ 5 Design and Analysis Software. Non-amplifying samples did not report C_q and were excluded from the data analysis. Amplifying samples with C_q over 35 were excluded from the data analysis because they did not achieve the PCR plateau phase. T_m was calculated with the proprietary QuantStudio™ 5 Design and Analysis Software based on the first derivative of fluorescence with respect to temperature. t_m was calculated from the second degree Savitsky–Golay polynomials[11] at each point (performed in MATLAB 2023A) based on the first derivative of fluorescence with respect to elapsed melt time. Salt concentration variability testing was evaluated using significant differences (Wilcoxon signed-rank test, significance level of α= 0.95) well-to-well of standard preparation as compared to salt-additive wild-type (n=2 trials with 36 replicates in both standard HRM and LHRM). The Wilcoxon signed-rank test was performed for T_m in standard HRM and for t_m and t_m difference in LHRM. All statistics were performed in Microsoft® Excel 2022.

L-DNA characterization

Table S8. Summary of D-DNA t_m's, L-DNA t_m's, and associated t_m differences for LHRM analyzed samples with double-stranded L-DNA at 1:1, 1:2, and 1:3 ratio of forward to reverse L-DNA strands (1×10^{11} forward strand copies and 1×10^{11}, 2×10^{11}, and 3×10^{11} reverse strand copies per reaction, respectively). L-DNA forward-to-reverse strand ratios shifted L-DNA t_m.

Ratio of Forward to Reverse L-DNA Strands	Average D-DNA t_m (sec±SD)	Average L-DNA t_m (sec±SD)	Average t_m Difference (sec±SD)
1:1	696.60±3.04	687.83±10.96	8.77±8.04
1:2	694.85±5.26	691.34±8.04	3.51±3.04
1:3	694.85±0.00	696.60±3.04	-1.75±3.04

Comparison of equal concentration 1:1 D-DNA and unlabeled 1:1 L-DNA

Table S9. Summary of T_m's and T_m differences for 1:1 D-DNA and 1:1 unlabeled L-DNA using three different intercalating dyes (n=1 trial in triplicate per sample type per intercalating dye). Unlabeled double-stranded L-DNA and D-DNA have small differences in melt temperatures measured by intercalation. The intercalating dyes did not discriminate between enantiomeric DNA.

Intercalating Dye	Average D-DNA T_m (ºC±SD)	Average L-DNA T_m (ºC±SD)	Average T_m Difference (ºC±SD)
EvaGreen®	82.81±0.14	82.44±0.00	0.38±0.14
EvaGreen® Plus	83.00±0.08	82.72±0.00	0.28±0.08
LCGreen® Plus	82.81±0.00	82.72±0.00	0.09±0.00

Early development of LHRM assumed that unlabeled wild-type L-DNA and D-DNA of equivalent sequence, length, strand ratio, and strand concentration would produce the same melt temperature. This assumption only holds if an intercalating dye works in the same way for right-handed and left-handed enantiomeric DNA stereoisomers. In practice, unlabeled wild-type L-DNA and D-DNA (**Table S10**) of equivalent sequence, length, and concentration actually produced different melt temperatures across all three intercalating dyes tested (**Table S9**). D-DNA T_m was higher than L-DNA T_m across all three intercalating dyes (**Table S9**). The average L-DNA to D-DNA T_m difference varied by intercalating dye: 0.38 ºC for EvaGreen®, 0.28 ºC for EvaGreen® Plus, and 0.09 ºC for LCGreen® Plus (**Table S9**). Although this data has not been previously reported in literature, the L-DNA versus D-DNA differences in melting behavior is speculated to be explained by intercalators fitting differently into left-handed versus right-handed enantiomeric DNA structures. Unique stereochemistry and shielded negative phosphate groups may alter intercalating dye mechanisms of action, thereby increasing L-DNA equivalent T_m. LCGreen® Plus was chosen as the intercalating dye of choice in LHRM because it presented the smallest T_m difference (0.09 ºC) amongst the dyes tested.

These studies were performed using wild-type *katG* sequence strands 56-bases in length. L-DNA was unlabeled to assess a true L-DNA versus D-DNA comparison of melting across three intercalators: EvaGreen®, EvaGreen® Plus, and LCGreen® Plus. Triplicates were prepared for each intercalating dye containing either 1:1 L-DNA or 1:1 D-DNA. Reactions were loaded into the 96-well plate such that each set of triplicates were in the same column, L-DNA samples were in the same rows as their D-DNA counterparts, and each set of intercalating dye samples were in mirrored locations across the left and right halves of the plate. Reactions had a 20 μL final volume containing 1X of SensiFAST™ Probe No-ROX Kit (Bioline #BIO-86005) and 1X of intercalating dye using either EvaGreen® Dye (Biotium #31000), EvaGreen® Plus Dye (Biotium #31077, or LCGreen® Plus (BioFire® Defense, LLC #BCHM-ASY-005). Each L-DNA reaction included 2 μL of L-DNA mix with final copy counts of 7.525×10^{11} copies unlabeled forward strand L-DNA (23FEB_katGf56) and 7.525×10^{11} copies unlabeled reverse strand L-DNA (23FEB_katGRcmp56) per reaction (**Table S10).** Each D-DNA reaction included 2 μL of D-DNA mix for final copy counts of 7.525×10^{11} copies forward strand D-DNA (MEP213) and 7.525×10^{11} copies reverse strand D-DNA (MEP214) per reaction (**Table S10**). A high resolution melt was performed by annealing 95 ºC to 50 ºC at 0.1 ºC/sec followed by melting 65 ºC to 95 ºC at 0.1 ºC/sec. Melt fluorescence was measured using the continuous acquisition mode and monitored using intercalating dye on the green optical channel (excitation 470±15 / emission 520±15). T_m was calculated with the proprietary QuantStudio™ 5 Design and Analysis Software based on the first derivative of fluorescence with respect to temperature. T_m difference was calculated as L-DNA T_m subtracted from D-DNA T_m in the well location mirrored across the 96-well plate.

Table S10. Oligonucleotide sequences designed for direct comparison of 1:1 L-DNA and unlabeled 1:1 D-DNA. DNA is denoted as D-DNA or L-DNA.

ID	DNA Type	Description	Sequence (5' → 3')
MEP213	D-DNA	Wild-type drug-susceptible amplicon forward strand	C ACC GGA ACC GGT AAG GAC GCG ATC ACC AGC GGC ATC GAG GTC GTA TGG ACG AAC A
MEP214	D-DNA	Wild-type drug-susceptible amplicon reverse strand	T GTT CGT CCA TAC GAC CTC GAT GCC GCT GGT GAT CGC GTC CTT ACC GGT TCC GGT G
23FEB_katGf56	L-DNA	Wild-type drug-susceptible internal comparator forward strand with end-labeling	C ACC GGA ACC GGT AAG GAC GCG ATC ACC AGC GGC ATC GAG GTC GTA TGG ACG AAC A
23FEB_katGRcmp56	L-DNA	Wild-type drug-susceptible internal comparator reverse strand with end-labeling	T GTT CGT CCA TAC GAC CTC GAT GCC GCT GGT GAT CGC GTC CTT ACC GGT TCC GGT G

PCR results

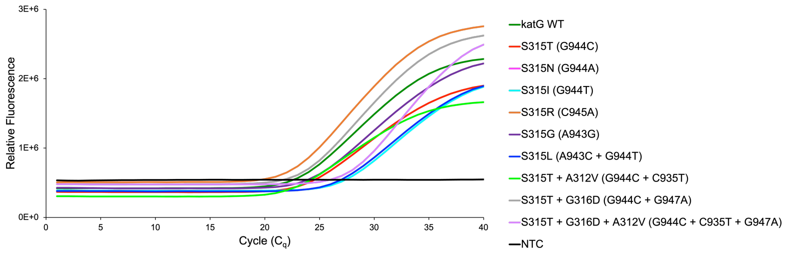

Figure S11. Representative PCR amplification curves of samples without L-DNA across wild-type (WT) *katG*, nine *katG* variants, and NTC sample types.

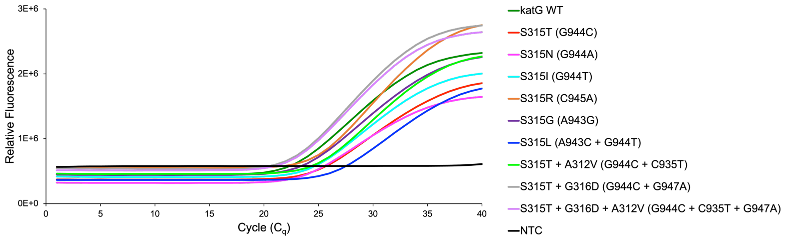

Figure S12. Representative PCR amplification curves of samples containing L-DNA across wild-type (WT) *katG*, nine *katG* variants, and NTC sample types.

ACKNOWLEDGEMENTS
We thank Logan Tsukiyama for preliminary investigational support. This work was supported in part by the National Institutes of Health (R21AI152497, R01AI157827, R01AI135937), the National Science Foundation Graduate Research Fellowship Program under Grant No. 1937963 awarded to N.A.M., and the National Science Foundation Graduate Research Fellowship Program under Grant No. 1937963 awarded to D.J.N. Any opinions, findings, and conclusions or recommendations expressed in this material are those of the author(s) and do not necessarily reflect the views of the National Science Foundation.

ADDITIONAL INFORMATION
Research Data is provided at https://github.com/nicolemalofsky/LHRM2024

REFERENCES
(1) Thermo Fisher Scientific Inc. *QuantStudio 3 and QuantStudio 5 Real-Time PCR Systems*; 2019. https://tools.thermofisher.com/content/sfs/brochures/quantstudio-3-and-5-real-time-pcr-systems.pdf (accessed 2023-09-10).
(2) Sun, L.; Wang, L.; Zhang, C.; Xiao, Y.; Zhang, L.; Zhao, Z.; Ren, L.; Peng, J. Rapid Detection of Predominant SARS-CoV-2 Variants Using Multiplex High-Resolution Melting Analysis. *Microbiol Spectr* **2023**, *11* (3). https://doi.org/10.1128/spectrum.00055-23.
(3) Bentaleb, E. M.; El Messaoudi, M. D.; Abid, M.; Messaoudi, M.; Yetisen, A. K.; Sefrioui, H.; Amzazi, S.; Ait Benhassou, H. Plasmid-Based High-Resolution Melting Analysis for Accurate Detection of RpoB Mutations in Mycobacterium Tuberculosis Isolates from Moroccan Patients. *BMC Infect Dis* **2017**, *17* (1). https://doi.org/10.1186/s12879-017-2666-4.
(4) Koshikawa, T.; Miyoshi, H. High-Resolution Melting Analysis to Discriminate between the SARS-CoV-2 Omicron Variants BA.1 and BA.2. **2022**. https://doi.org/10.1016/j.bbrep.2022.101306.
(5) Miyoshi, H.; Ichinohe, R.; Koshikawa, T. High-Resolution Melting Analysis after Nested PCR for the Detection of SARS-CoV-2 Spike Protein G339D and D796Y Variations. **2022**. https://doi.org/10.1016/j.bbrc.2022.03.083.
(6) Thermo Fisher Scientific Inc. QuantStudio 3 and 5 Real-Time PCR Systems Installation, Use, and Maintenance Guide. **2021**.
(7) Hristea, A.; Otelea, D.; Paraschiv, S.; MacRi, A.; Baicus, C.; Moldovan, O.; Tinischi, M.; Arama, V.; Streinu-Cercel, A. Detection of Mycobacterium Tuberculosis Resistance Mutations to Rifampin and Isoniazid by Real-Time PCR. *Indian Journal of Medical Microbiology*. Medknow Publications and Media Pvt. Ltd July 1, 2010, pp 211–216. https://doi.org/10.4103/0255-0857.66474.
(8) Liu, Z. bin; Cheng, L. ping; Pan, H. qiu; Wu, X. cui; Lu, F. hui; Cao, J.; Wang, L.; Wei, W.; Chen, H. yu; Sha, W.; Sun, Q. Performance of the MeltPro TB Assay as Initial Test for Diagnosis of Pulmonary Tuberculosis with Drug-Resistance Detection. *Molecular Medicine* **2023**, *29* (1). https://doi.org/10.1186/s10020-023-00743-1.
(9) Thant, Y. M.; Saikaew, S.; Tharinjaroen, C. S.; Phunpae, P.; Pongsararuk, R.; Preechasuth, K.; Butr-Indr, B.; Intorasoot, S.; Tragoolpua, K.; Chaiprasert, A.; Wattananandkul, U. Combined Locked Nucleic Acid Probes and High-Resolution Melting Curve Analysis for Detection of Rifampicin-Resistant Tuberculosis in Northern Thailand. *Diagnostics* **2022**, *12* (10). https://doi.org/10.3390/diagnostics12102307.
(10) Anderson, D. J.; Reischer, R. J.; Taylor, A. J.; Wechter, W. J. Preparation and Characterization of Oligonucleotides of D- and L-2' Deoxyuridine. *http://dx.doi.org/10.1080/07328318408081285* **2007**, *3* (5), 499–512. https://doi.org/10.1080/07328318408081285.
(11) Wittwer, C. T.; Reed, G. H.; Gundry, C. N.; Vandersteen, J. G.; Pryor, R. J. High-Resolution Genotyping by Amplicon Melting Analysis Using LCGreen. *Clin Chem* **2003**, *49* (6), 853–860. https://doi.org/10.1373/49.6.853.
(12) Youden, W. J. Index for Rating Diagnostic Tests. *Cancer* **1950**, *3* (1), 32–35. https://doi.org/10.1002/1097-0142(1950)3:1<32::AID-CNCR2820030106>3.0.CO;2-3.
(13) Liew, M.; Pryor, R.; Palais, R.; Meadows, C.; Erali, M.; Lyon, E.; Wittwer, C. Genotyping of Single-Nucleotide Polymorphisms by High-Resolution Melting of Small Amplicons. *Clin Chem* **2004**, *50* (7), 1156–1164. https://doi.org/10.1373/clinchem.2004.032136.
(14) Słomka, M.; Sobalska-Kwapis, M.; Wachulec, M.; Bartosz, G.; Strapagiel, D. High Resolution Melting (HRM) for High-Throughput Genotyping-Limitations and Caveats in Practical Case Studies. *International Journal of Molecular Sciences*. MDPI AG November 3, 2017. https://doi.org/10.3390/ijms18112316.
(15) Urata, H.; Shinohara, K.; Ogura, E.; Yoshiaki, U.; Akagi, M. Mirror-Image DNA. *J. Am. Chem. Soc.* **1991**, *113*, 8174–8175.
